# Classification of gallstones using Fourier-transform infrared spectroscopy and photography

**DOI:** 10.1186/s40824-018-0128-8

**Published:** 2018-07-18

**Authors:** Byeong Jo Ha, Sangsoo Park

**Affiliations:** 1 Department of Beauty and Cosmetics, College of Health Science, Eulji University, Seongnam, Gyeonggi-do 13135 South Korea; 2 Department of Biomedical Engineering, College of Health Science, Eulji University, 553 Sanseongdae-ro, Sujeong-gu, Seongnam, Gyeonggi-do 13135 Republic of Korea

**Keywords:** Gallstone composition, FT-IR spectroscopy, Cholesterol, Calcium bilirubinate, Calcium carbonate

## Abstract

**Background:**

Gallstones have conventionally been classified by gross inspection into 4 categories: cholesterol gallstones, black pigment (calcium bilirubinate) gallstones, brown gallstones, and mixed gallstones that contain both cholesterol and calcium bilirubinate. Classification using Fourier-transform infrared (FT-IR) spectroscopy supplements gross inspection; however, the issue of ambiguity in gallstone classification has not been fully addressed to date.

**Methods:**

Twenty-six gallstones obtained after surgical gallbladder removal were examined using FT-IR spectroscopy and digital photography, and classified into 6 gallstone groups according to characteristic FT-IR absorption bands.

**Results:**

FT-IR spectra of nine gallstones matched well with that of pure cholesterol, and the gallstones were thus classified as cholesterol stones. Twelve gallstones were classified as calcium bilirubinate stones as they showed characteristic absorption bands of calcium bilirubinate. However, the FT-IR spectra of these gallstones always showed a broad absorption band of bound water at 3600–2400 cm^− 1^. The other five gallstones were classified as mixed stones with combinations of cholesterol, calcium bilirubinate, and calcium carbonate.

**Conclusion:**

FT-IR spectroscopy is a powerful and convenient method for gallstone classification. Nevertheless, one should take serious note of the superposition of FT-IR absorption bands of different chemical components of gallstones including that of bound water.

## Background

Gallstone formation in the gallbladder, bile duct, and liver is a common digestive disease, occurring in 10–20% of the population in Western countries, and approximately 25% of these patients eventually require surgical removal due to severe symptoms [1, 2]. Traditionally, gallstones have been divided by gross inspection into 4 categories: cholesterol stones, black pigment (calcium bilirubinate) stones, brown color stones, and mixed stones that consist of both cholesterol and calcium bilirubinate [2–4]. However, this classification method is largely dependent upon the external shape and color of gallstones and does not accurately reflect the cases wherein the internal morphology of gallstones is different from the external one.

Fourier-transform infrared (FT-IR) spectroscopy has been a major tool for gallstone classification and constituent analysis [5–13], as the technique is fast and applicable to all types of gallstones, irrespective of crystallinity, and requires only a small amount of sample. FT-IR is particularly useful when gallstone samples from a large number of patients are examined for purposes of classification and studying the etiology of gallstone formation [5–9]. It is now well established through these studies that cholesterol, calcium bilirubinate, and calcium carbonate are the three major constituents of gallstones. For detailed examination of gallstone constituents, fluorescence microscopy [13], X-ray diffraction [14–17], thermogravimetry (TG) and difference scanning calorimetry (DSC) [18], specular reflection spectroscopy [19], scanning electron microscopy (SEM), energy-dispersive X-ray spectroscopy (EDX), and Nuclear Magnetic Resonance (NMR) have been applied for selected samples [20].

FT-IR has contributed greatly to our understanding of gallstone composition and is the major tool of choice for classification of gallstones. Nevertheless, together with visual inspection, gallstone classification using FT-IR is far from being unambiguous, as most gallstones are a mixture of several chemical substances and the absorption bands of chemical species often overlap. In this paper, we report the results of classification of 26 gallstones by FT-IR and photography, and address the ambiguity issues of gallstone classification.

## Methods

Twenty-six gallstones, collected from gallbladders after cholecystectomy, were provided by the department of internal medicine at Eulji University Hospital without any patient information. After washing with deionized water, the gallstones were dried under vacuum for 12 h. Once the drying process was completed, a digital photograph was taken of each gallstone. The gallstone was halved by a scalpel for a digital photograph of the internal morphology. The gallstone was then powdered using an agate mortar and pestle. The powder was diluted with KBr in a proportion of 1% (*w*/w). By using a special dye, the powder was pressed on to a translucent film, which was subsequently used for the analysis by a FT-IR spectrophotometer (FTS 3000, Bio-Rad, Cambridge, MA, USA) at 400–4000 cm^− 1^ with a 4 cm^− 1^ resolution. Each sample was subject to 100 scans and the average spectrum was obtained in the absorbance mode.

The gallstones were classified by comparing the respective absorption peaks with the values reported in the literature, as shown in Table 1. The photographs of gallstones from each group were collected and compared with each other to identify the presence of a common morphology in the same group.

**Table 1:** Characteristic FT-IR absorption bands of 3 major gallstone constituents, cm^− 1^

	Laloum et al. [12]	Kleiner et al. [13]	Gang et al. [19]	Suo et al. [18]	Our study
Cholesterol	1467, 1378, 1058	3398, 2933, 2866 1463, 1376, 1056	3408, 2934, 2867 1446, 1367, 1057	3395, 2930, 2867 1464, 1374, 1056	3410, 2934, 2853, 1458, 1373, 1055
Calcium bilirubinate	1666, 1628, 1571, 1251	1661, 1640, 1575	3402, 1696, 1663, 1620, 1572, 1448, 1250, 700	1700, 1662, 1628, 1574, 1460, 1253, 1054	3398, 1663, 1624, 1566, 1447, 1251, 699
Calcium carbonate	1480, 1419 875, 855	1464, 875		broad band at 1300–1500, 875, 711	1464, 1458, 1420, 872, 855

## Results

Twenty-six gallstones were classified into six groups after careful examination of each gallstone’s FT-IR spectrum, as shown in Table 2.

**Table 2:** Number and percentage of gallstone groups determined by FT-IR^a^

	Cholesterol gallstones			Calcium bilirubinate gallstones				
	Pure	+ CaCO_3_	+ CaCO_3_ + CB	Pure	+CP	+ Chol	+ CaCO_3_	+ Chol + CPH
Laloum et al. [14]	53 (41.1)	0	6 (4.7)	26 (20.1)	20 (15.5)	5 (3.9)	8 (6.2)	11 (8.5)
Our study	9 (34.6)	1 (3.8)	1 (3.8)	12 (42.3)	0	1 (3.8)	2 (7.6)	0

^a^*Chol* Cholesterol, *CaCO*_*3*_ calcium carbonate, *CB* Calcium bilirubinate, *CP* calcium palmitate, *CPH* Calcium phosphate

### Pure cholesterol gallstones

**Fig. 1:**
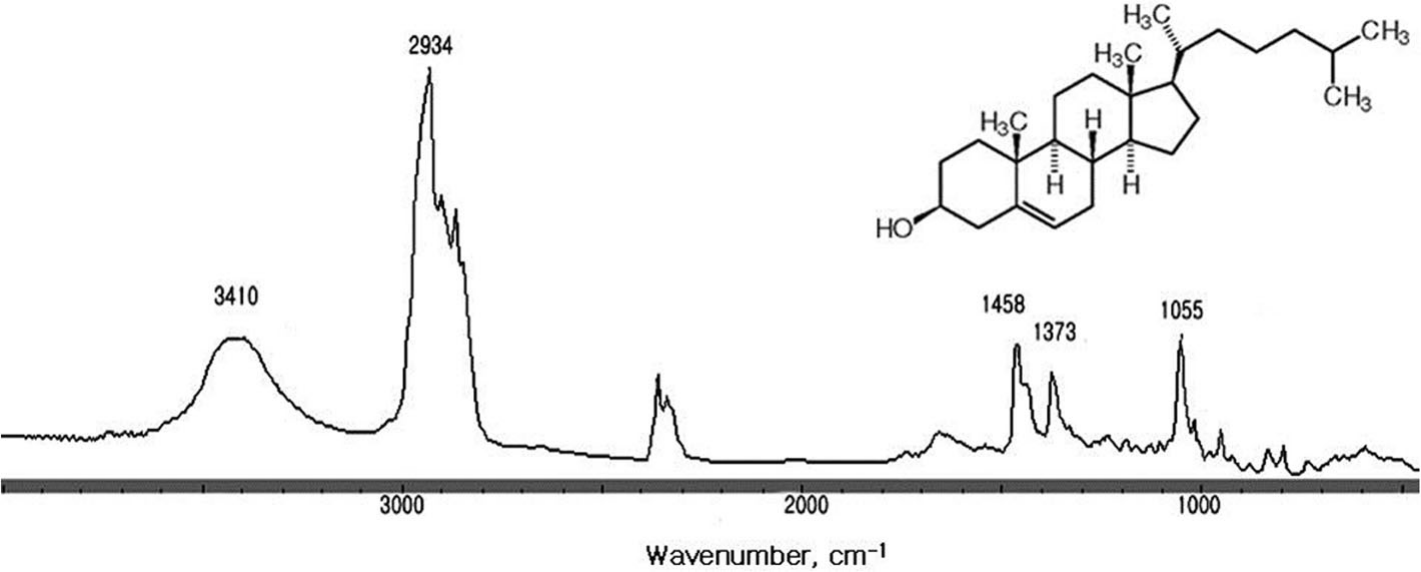
Chemical structure of cholesterol and typical Fourier-transform infrared (FT-IR) spectrum of a pure cholesterol gallstone. The broad absorption band of cholesterol OH group is centered at 3410 cm^− 1^

The FT-IR spectrum of a pure cholesterol gallstone as well as the chemical structure of cholesterol is shown in Fig. 1, wherein y axis unit is absorbance and the axis is removed for display. Table 1 shows the comparison between the FT-IR absorption peaks of cholesterol gallstones in this study and those reported in previous studies. The FT-IR spectrum of a cholesterol gallstone was composed of a CH_2_ asymmetric stretching absorption band at 2934 cm^− 1^, a CH_2_ symmetric stretching absorption band at 2860 cm^− 1^, a CH_2_ asymmetric bending absorption band at 1458 cm^− 1^, a CH_2_ symmetric bending absorption band at 1373 cm^− 1^, and a C-C stretching absorption band at 1055 cm^− 1^ [10] A broad OH stretching mode absorption band was centered at 3410 cm^− 1^. The band at 2353 cm^− 1^ was an artifact representing the CO_2_ absorption by air. There was no other absorption peak observed in this spectrum, indicating that these stones were composed of pure cholesterol. Of the 26 gallstones, nine (34.6%) belonged to the pure cholesterol gallstone group.

**Fig. 2:**
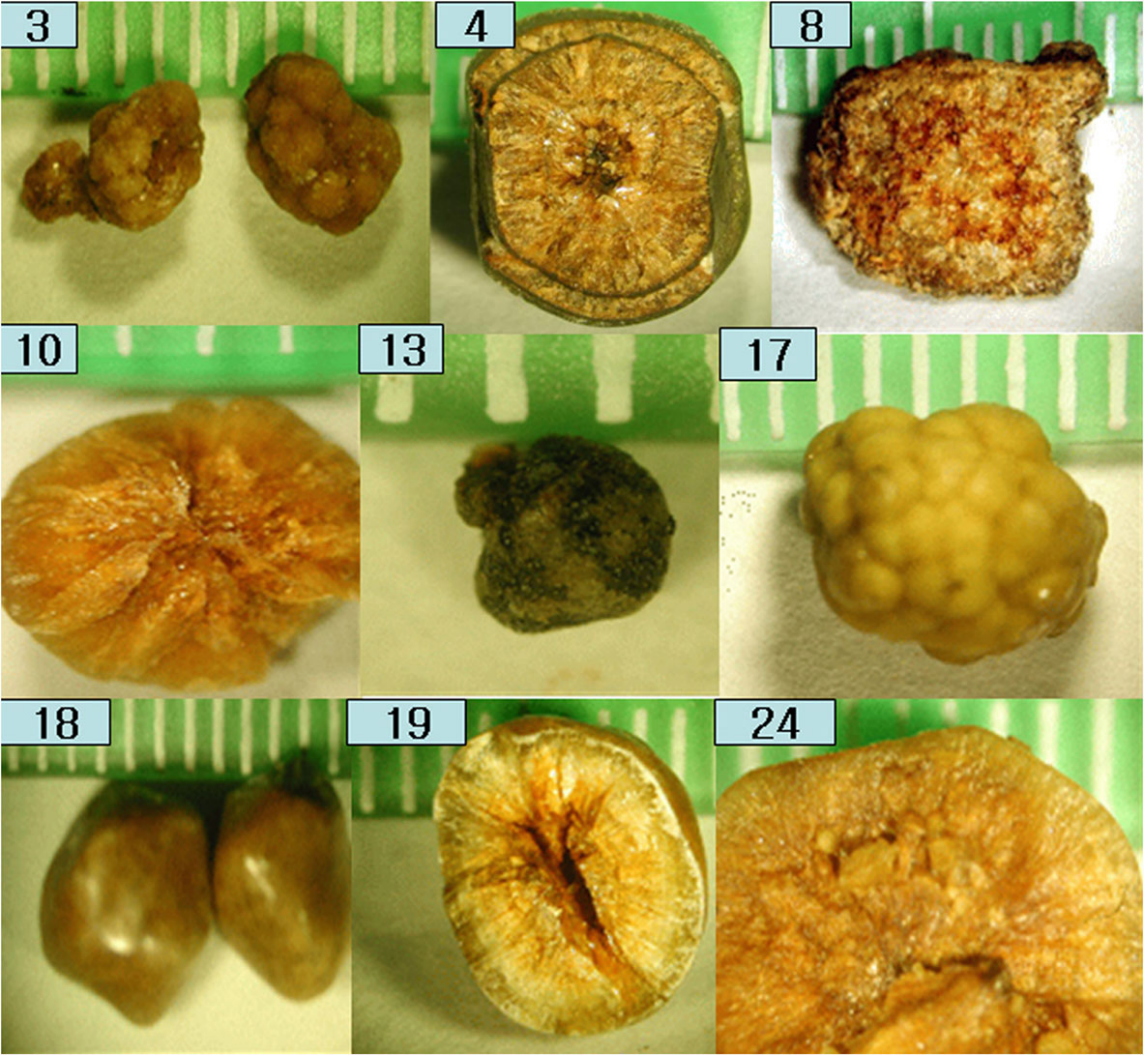
Photographs of pure cholesterol gallstones. Three stones (#10, #17, #24) are completely yellow inside and out, but the other 6 gallstones are covered with a green or brown layer

The photographs of pure cholesterol gallstones are collected in Fig. 2, and a green plastic ruler demonstrated the dimensions of the stone. Three stones were multiple and the remaining six were singular. Two were of multinuclear berry type (#3, #17). Three stones (#10, #17, #24) were completely yellow inside and out, but the surface of the other six gallstones was covered with either a green or brown layer.

### Calcium bilirubinate gallstones

**Fig. 3:**
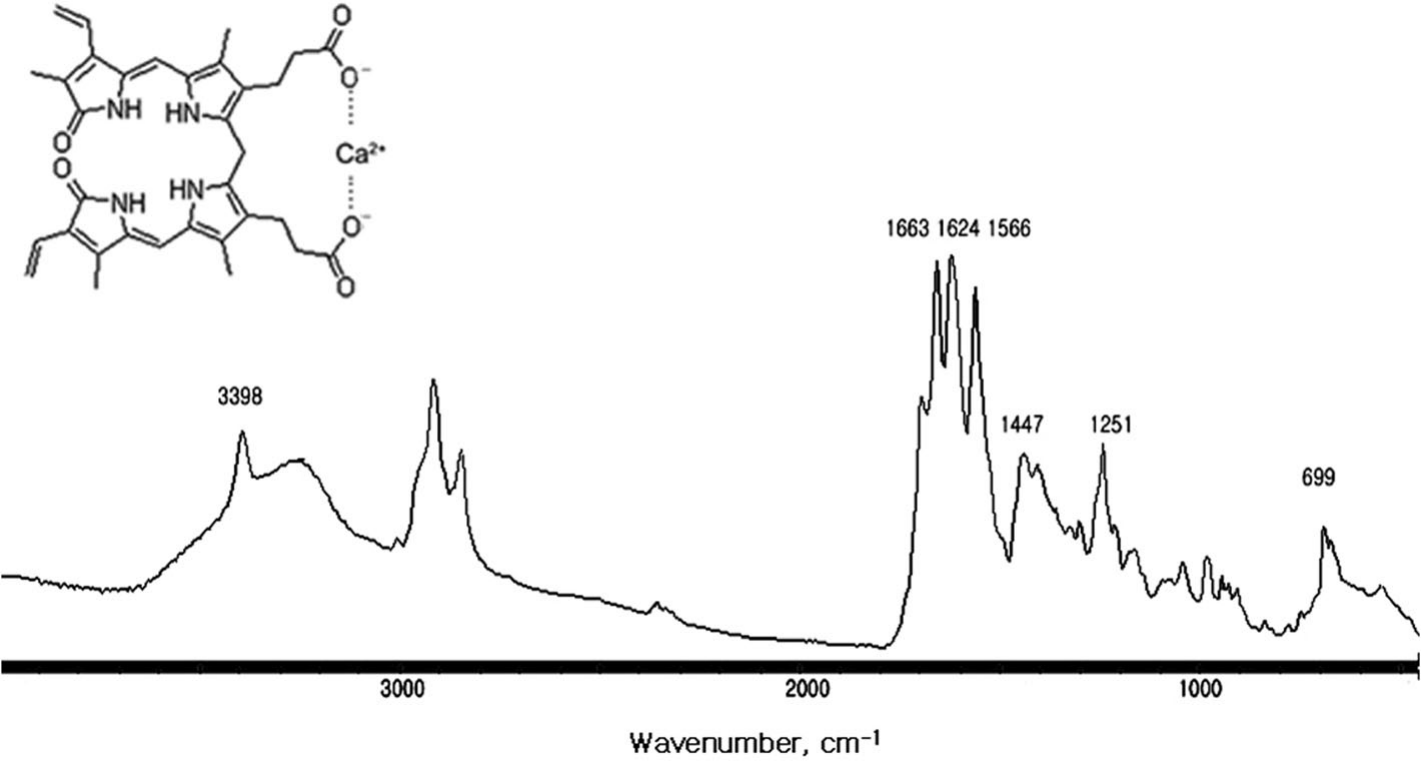
Chemical structure of calcium bilirubinate and Fourier-transform infrared (FT-IR) spectrum of a calcium bilirubinate gallstone. The spectrum has a characteristic triplet absorption peak centered at around 1624 cm^− 1^ and a pyrrole N-H absorption peak at 3398 cm^− 1^

The FT-IR spectrum of a calcium bilirubinate gallstone, together with chemical structure of calcium bilirubinate is shown in Fig. 3, wherein y axis unit is absorbance and the axis is removed for display. The spectrum matched well with the FT-IR spectrum of pure calcium bilirubinate reported by Suo et al. [19]. The spectrum had characteristic doublet absorption peaks at 3398 cm^− 1^ and 3264 cm^− 1^ as well as triplet peaks at 1663, 1624, and 1566 cm^− 1^. One of the doublet absorption peaks at 3398 cm^− 1^ is sharp, and the band at 3264 cm^− 1^ was broad and it appeared that the latter overlapped with a broad absorption band at 3600–2400 cm^− 1^. The sharp absorption peak at 3398 cm^− 1^ was previously assigned as a N-H stretching vibration of the pyrrole groups, and the broad absorption band at 3264 cm^− 1^ as the lactam N-H stretching vibration [18]. The broad absorption band at 3600–2400 cm^− 1^ was identified as the absorption of bound water, H_2_O in Ca(HBR)_2_.H_2_O or Ca(Br).2H_2_O, where HBR and BR stand for monovalent and divalent bilirubinate, respectively [19]. The absorption peak at 3398 cm^− 1^ was clearly separated from the band at 3264 cm^− 1^ for six out of the 12 gallstones, owing to the broad absorption band of bound water. The absorption band at 3264 cm^− 1^ appeared as a shoulder of the absorption peak at 3398 cm^− 1^ for five other gallstones. With respect to triplet absorption peaks, 1663 and 1624 cm^− 1^ peaks were observed for bilirubin, and the 1566 cm^− 1^ band was observed only when the carboxylic acid of bilirubin is conjugated with a metal ion such as calcium, forming calcium bilirubinate [8]. In addition, a shoulder peak observed at 1703 cm^− 1^ was assigned to the vibration of a non-conjugated carboxylic acid [12, 19]. The presence of the absorption peak at 1703 cm^− 1^ indicated that some carboxylic acid groups in the gallstone are monovalent, i.e., calcium bilirubinate in the gallstone includes both Ca(HBR)_2_.H_2_O and Ca(Br) .2H_2_O forms. The absorption bands at 1447 cm^− 1^ were assigned to the pyrrole ring deformation and were observed for both bilirubin and calcium bilirubinate. Furthermore, the absorption peak at 1251 cm^− 1^ was previously assigned for amino C-N/carboxylate C-O stretching, and at 699 cm^− 1^ for the lactam ring deformation. FT-IR spectrum of pure calcium bilirubinate, reported by Suo et al., had a doublet absorption band at above 3000 cm^− 1^ and triplet absorption peaks between 1500 and 1700 cm^− 1^, without the broad absorption band of bound water at 3600–2400 cm^− 1^.

**Fig. 4:**
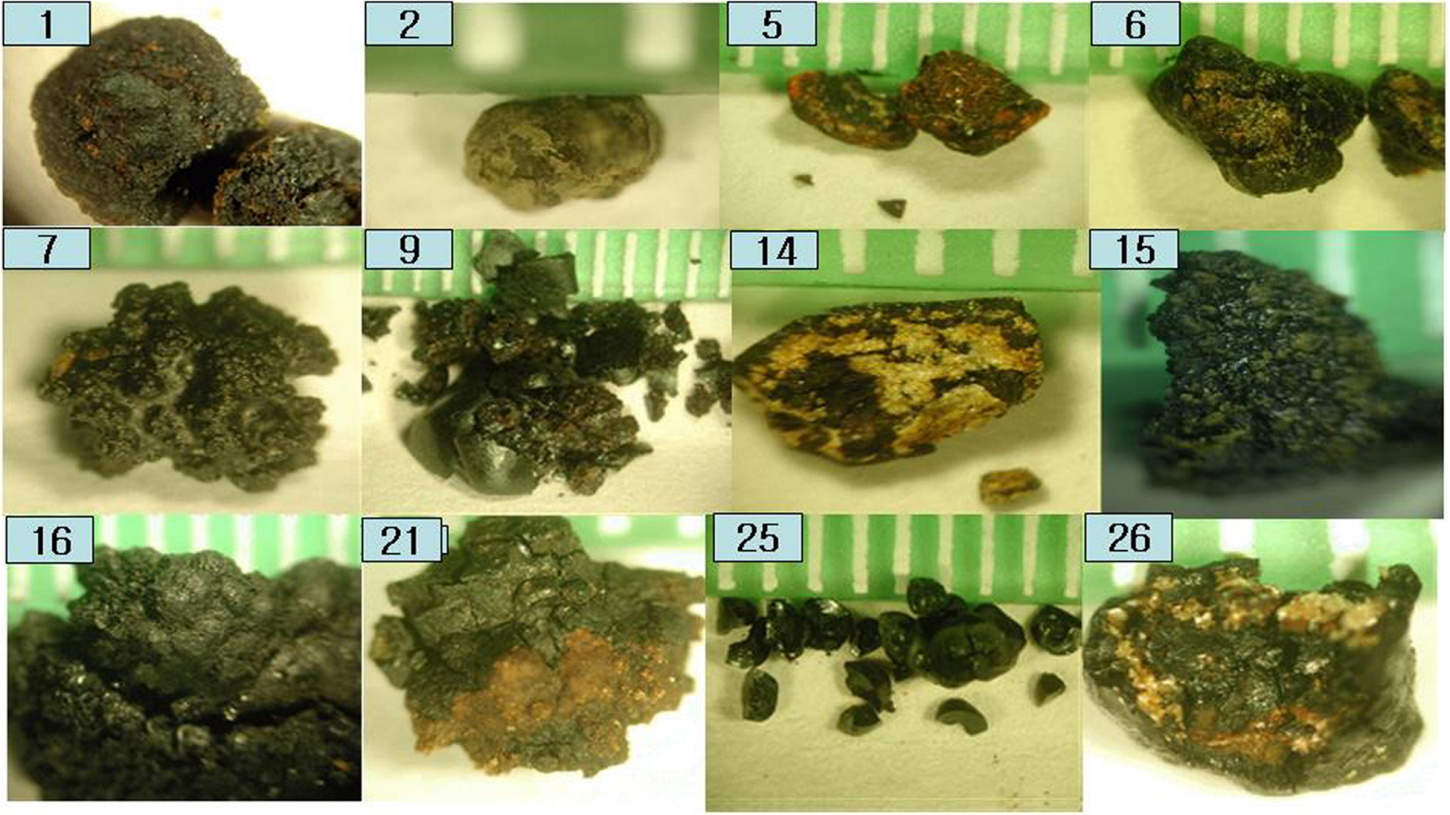
Photographs of calcium bilirubinate gallstones. The gallstones are mostly black but some include a reddish yellow substance

The photographs of the gallstones belonging to the calcium bilirubinate gallstone group are shown in Fig. 4. Twelve out of the 26 gallstones belonged to this group (46.0%). Calcium bilirubinate gallstones were predominantly black, but gallstone #14 had some yellow-colored substance. The FT-IR spectrum did not show the characteristic absorption peaks of cholesterol (CaCO_3_ or Ca_3_PO_4_), and hence, we suspected that the yellow substance was a chemical species with a small absorption coefficient.

### Cholesterol gallstones mixed with calcium carbonate or calcium bilirubinate

**Fig. 5:**
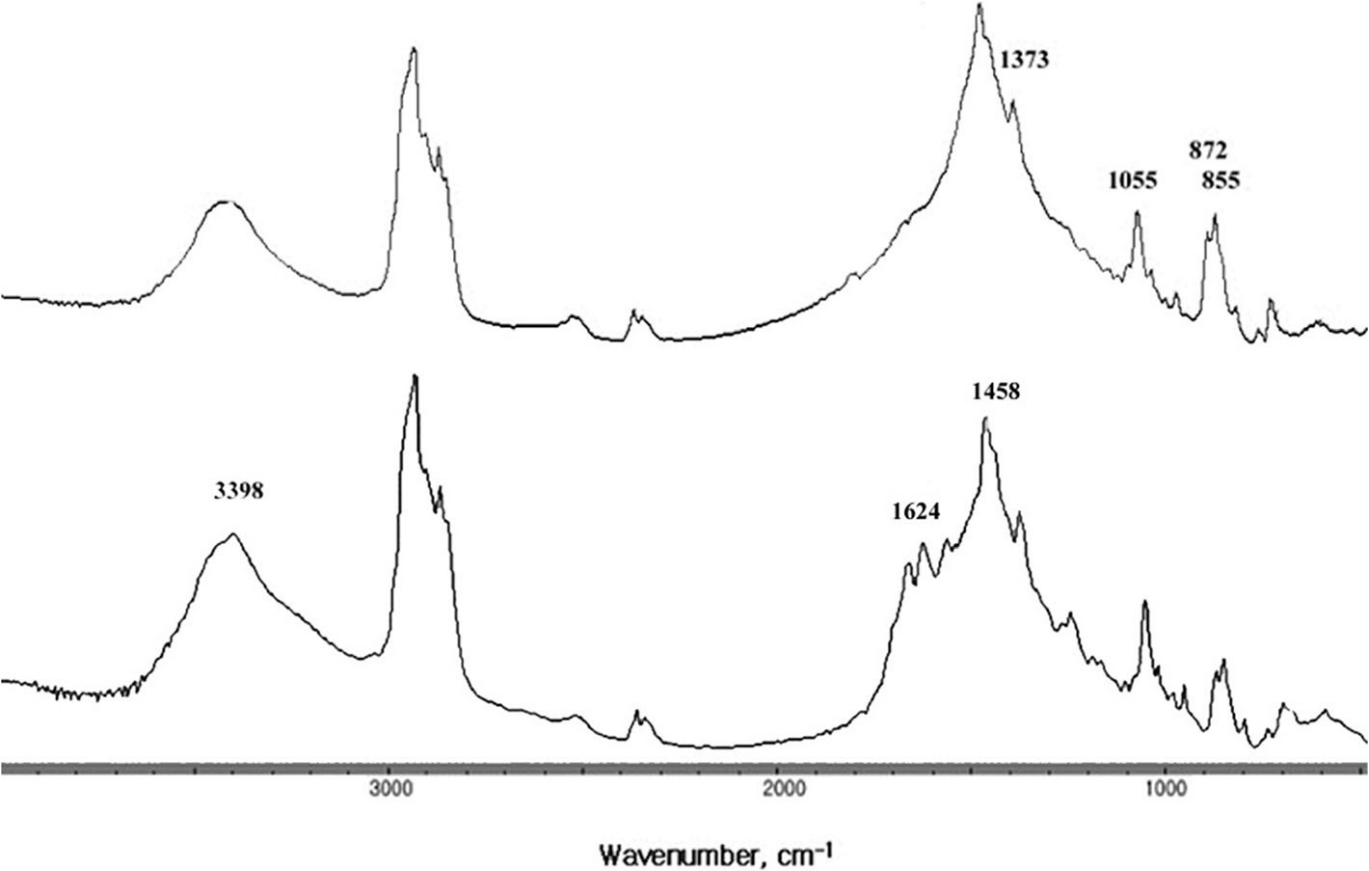
Fourier-transform infrared (FT-IR) spectra of a cholesterol gallstone mixed with calcium carbonate (top) and a cholesterol gallstone mixed with both calcium carbonate and calcium bilirubinate (bottom). The presence of calcium carbonate is indicated by the absorption peaks at 1458, 872, and 855 cm^− 1^, and calcium bilirubinate by a triplet absorption peak centered at 1624 cm^− 1^ and a pyrrole N-H absorption peak at 3398 cm^− 1^

**Fig. 6:**
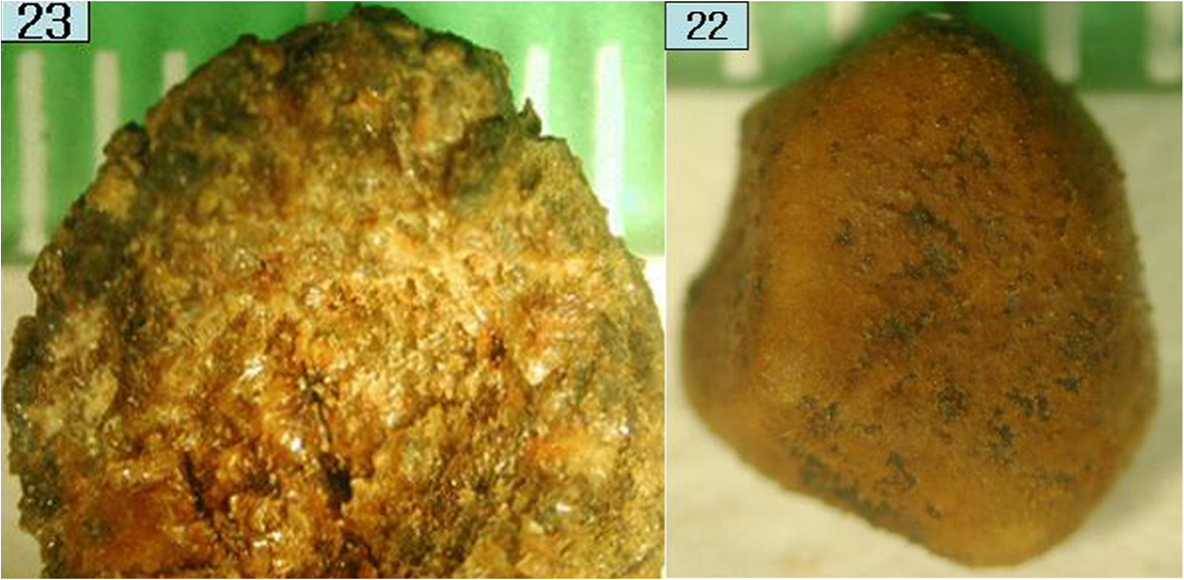
Photographs of a cholesterol gallstone mixed with calcium carbonate (#23) and a cholesterol gallstone mixed with both calcium carbonate and calcium bilirubinate (#22)

The FT-IR spectra of cholesterol stones mixed with calcium carbonate or calcium bilirubinate are collected and displayed in Fig. 5, wherein y axis unit is absorbance and. The y axis was offset and removed for display and comparison. The FT-IR spectrum of calcium carbonate is known to have a broad absorption peak at 1420–1480 cm^− 1^, as well as sharp absorption peaks at 872 and 855 cm^− 1^ [14, 15]. The FT-IR spectrum of a cholesterol stone mixed with calcium carbonate consisted of the characteristic absorption bands of cholesterol at 3410, 2933, and 1055 cm^− 1^. However, the asymmetric bending mode absorption peak of cholesterol CH_2_ at 1458 cm^− 1^ was superimposed on the broad absorption band of CO_3_^2−^ ion and the resultant peak had a higher intensity than that of the pure cholesterol gallstone, as shown in Fig. 1. The CH_2_ symmetric bending mode peak of cholesterol at 1373 cm^− 1^ appeared as a shoulder of the carbonate absorption band at 1464 cm^− 1^. In addition, the characteristic absorption peak of carbonate at 872 and 855 cm^− 1^ was observed for cholesterol gallstones mixed with calcium carbonate. Only one gallstone belongs to this group (3.8%), with a morphology similar to that of a pure cholesterol gallstone, but the cut surface was neither radial nor concentric; it was irregular and glossy (#23 in Fig. 6).

**Fig. 7:**
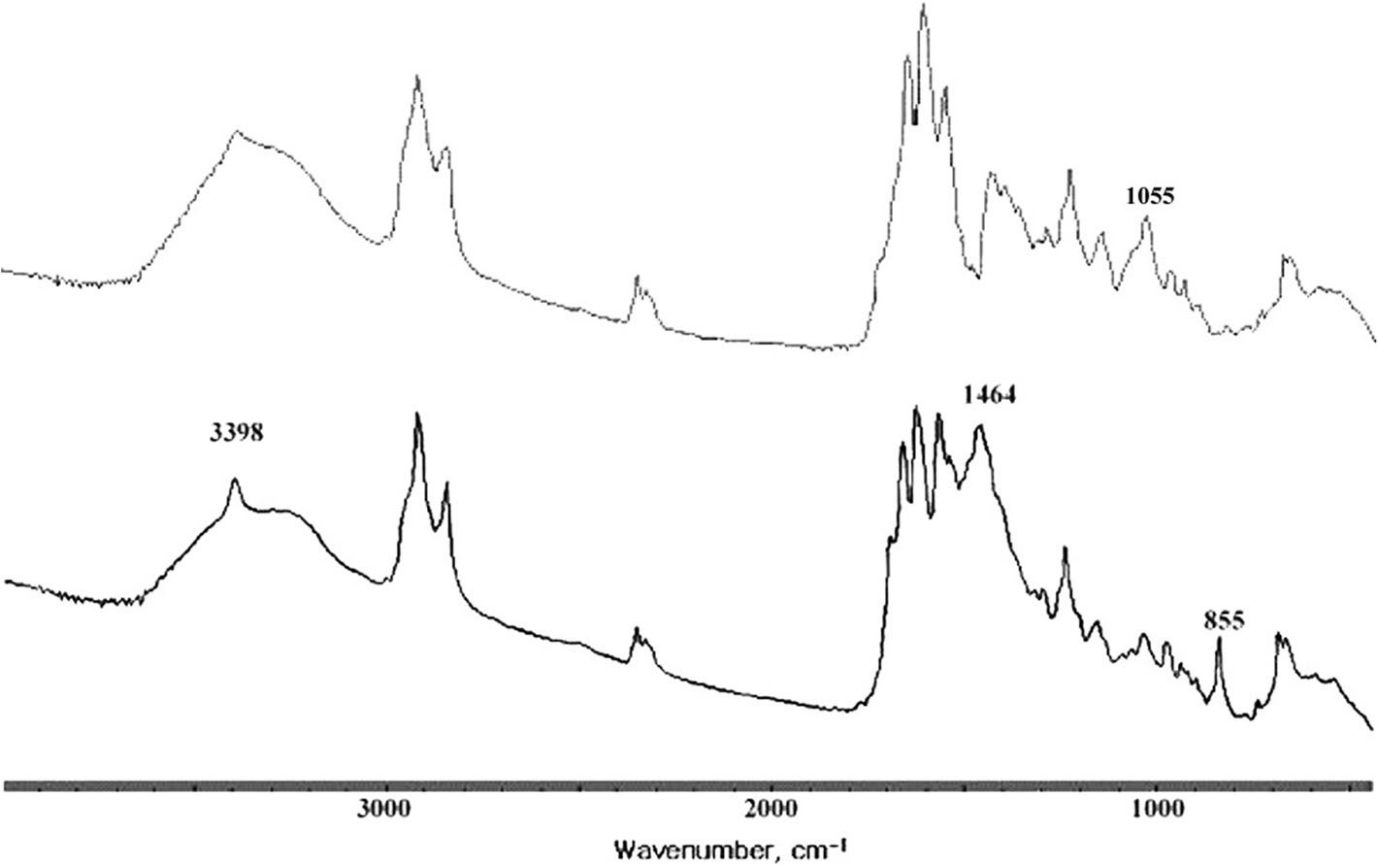
Fourier-transform infrared (FT-IR) spectra of calcium bilirubinate gallstone mixed with cholesterol (top) and a calcium bilirubinate gallstone mixed with calcium carbonate (bottom)

The FT-IR spectrum of a cholesterol gallstone mixed with calcium bilirubinate together with calcium carbonate, shown in Fig. 7, had a typical triplet absorption band of a calcium bilirubinate centered at 1624 cm^− 1^ as well as a typical calcium carbonate broad absorption band centered at 1458 cm^− 1^. The absorption band at 3410 cm^− 1^ shifts to 3398 cm^− 1^ as the cholesterol OH absorption band overlapped with the pyrrole absorption band of calcium bilirubinate. The absorption bands at 1458, 872, and 855 cm^− 1^ were indications of the presence of a carbonate CO_3_^2−^. Only one gallstone belonged to this group (3.8%). This gallstone appears as a multi-faceted brown color stone (#22 in Fig. 6).

### Calcium bilirubinate gallstone mixed with cholesterol

**Fig. 8:**
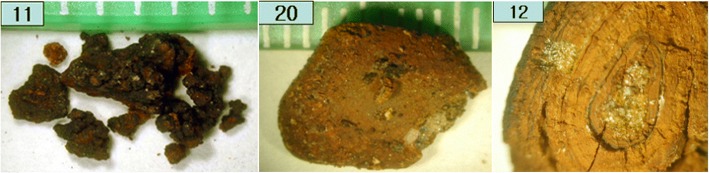
Photographs of calcium bilirubinate gallstones mixed with calcium carbonate (#11, #20) and a calcium bilirubinate gallstone mixed with cholesterol (#12)

As shown in Fig. 7, the FT-IR spectrum of a calcium bilirubinate gallstone mixed with cholesterol had all the characteristic absorption peaks of calcium bilirubinate (3398, 2919, 1663, 1624, 1566, 1251, and 699 cm^− 1^). In addition, it had a characteristic cholesterol absorption peak at 1055 cm^− 1^. One gallstone belonged to this group (3.8%). This gallstone had a radial and concentric morphology and was brown in color (#12 in Fig. 8).

### Calcium bilirubinate gallstone mixed with calcium carbonate

The FT-IR spectrum of a calcium bilirubinate gallstone mixed with calcium carbonate is shown in Fig. 7, wherein y axis unit is absorbance. The y axis was offset and removed for display and comparison. The spectrum had all the characteristic calcium bilirubinate absorption bands as well as characteristic calcium carbonate (calcite) absorption peaks at 1464, 855 cm-1. These gallstones were brownish in color and contained scattered inorganic particles inside and out (#11 and #20 in Fig. 8). Two gallstones belonged to this group (7.7%).

## Discussion

We studied the FT-IR spectra and photographic images of 26 gallstones and classified them into six categories based upon the FT-IR absorption characteristics. Institutional review for gallstone collection was not necessary, as the gallstones were the remnants of cholecystectomy (resection of gallbladder for treatment of acute cholecystitis). According to Article 33 of the Enforcement Rule of Bioethics and Safety Act by the Korean government, institutional review can be exempt for this type of research as long as patient information is withheld.

Characteristic absorption bands of the three major components of the gallstones, namely cholesterol, calcium bilirubinate, and calcium carbonate, corresponded to the reported values well within the ranges of experimental error, as shown in Table 1. All the pure cholesterol gallstones showed a consistent FT-IR spectrum and gallstones with a very distinct green or brown surface color did not show any significant FT-IR absorption band other than that of cholesterol. This indicates that the green or brown surface layer was very thin, as can be confirmed from the photographs in Fig. 1.

The highest number of gallstones, eleven out of a total of twenty-six (46.0%), belonged to the calcium bilirubinate group, although they are black in most cases, some have red or yellow particles scattered inside and out. However, there were no significant FT-IR absorption bands indicating cholesterol, calcium carbonate, or calcium phosphate. This could be due to a drawback of FT-IR technique: FT-IR absorption is not sensitive to minor chemical species, particularly when the minor component has a smaller extinction coefficient than the major component. Other analytical techniques should have been applied for identification of these particles, but we mistakenly powdered the whole gallstone for preparing the FT-IR sample. We learned a valuable lesson that a part of the gallstone should be saved for further analysis, especially if the gallstone has a visually apparent second component.

Calcium carbonate in a gallstone is easy to identify using FT-IR spectroscopy as the characteristic absorption bands at 855 and 872 cm^− 1^ do not interfere with the absorption bands of cholesterol or calcium bilirubinate. Calcium carbonate can be found together with cholesterol, calcium bilirubinate, or both calcium bilirubinate and cholesterol, and the color of these gallstones are light to dark brown in all cases, as can be seen in Figs. 6 and 8.

Gallstones have traditionally been classified as cholesterol, black pigment, brown, and mixed, based primarily on external color and morphology [1–3]. It should be noted, however, that a pure cholesterol gallstone could occasionally be misjudged as a brown gallstone due to a very thin brown-colored layer covering the stone, as seen in Fig. 2. Most black gallstones were revealed to be calcium bilirubinate gallstones. Brown gallstones should be classified with care, as they could be a cholesterol gallstone covered with a very thin brown layer (Fig. 2), a mixed gallstone of cholesterol with calcium bilirubinate and calcium carbonate (Fig. 6), or a mixed gallstone of calcium bilirubinate with calcium carbonate (Fig. 8).

A side-by-side comparison of the FT-IR spectrum of a gallstone with its corresponding photographic image was attempted previously for selected gallstone samples, and it helped to understand the chemical composition and morphology of those gallstones. To our knowledge, however, this study is the first attempt to compare and classify gallstones using the FT-IR spectra and photographic images of the whole gallstone samples in a gallstone classification study.

## Conclusion

FT-IR has been tool of choice for classifying a large number of gallstone samples. However, this study demonstrated that care must be taken for classifying the gallstones using FT-IR alone as the absorption bands of gallstone constituents often overlap with each other and the absorption of bound water makes the interpretation of FT-IR spectrum of calcium bilirubinate gallstones difficult. Interpretation of the FT-IR spectrum of a gallstone should be given due attention, and further research using various analytical techniques is warranted for understanding the constituents of gallstones and the etiology of gallstone formation.

## Abbreviation

FT-IR: Fourier-transform infrared
